# Crafting a prognostic nomogram for the overall survival rate of cutaneous verrucous carcinoma using the surveillance, epidemiology, and end results database

**DOI:** 10.3389/fendo.2023.1142014

**Published:** 2023-03-27

**Authors:** Siomui Chong, Liying Huang, Hai Yu, Hui Huang, Wai-kit Ming, Cheong Cheong Ip, Hsin-Hua Mu, Kexin Li, Xiaoxi Zhang, Jun Lyu, Liehua Deng

**Affiliations:** ^1^ Department of Dermatology, The First Affiliated Hospital of Jinan University and Jinan University Institute of Dermatology, Guangzhou, China; ^2^ Department of Clinical Research, The First Affiliated Hospital of Jinan University, Guangzhou, China; ^3^ Department of Infectious Diseases and Public Health, Jockey Club College of Veterinary Medicine and Life Sciences, City University of Hong Kong, Hong Kong, Hong Kong SAR, China; ^4^ Department of Dermatology, University Hospital Macau, Macau, Macao SAR, China; ^5^ General Surgery Breast Medical Center, Taipei Medical University Hospital, Taipei, China; ^6^ Department of Dermatology, The Fifth Affiliated Hospital of Jinan University, He Yuan, China; ^7^ Guangdong Provincial Key Laboratory of Traditional Chinese Medicine Informatization (2021B1212040007), Guangzhou, China

**Keywords:** cutaneous verrucous carcinoma, nomograms, SEER, prognosis, overall survival (OS)

## Abstract

**Background:**

The aim of this study was to establish and verify a predictive nomogram for patients with cutaneous verrucous carcinoma (CVC) who will eventually survive and to determine the accuracy of the nomogram relative to the conventional American Joint Committee on Cancer (AJCC) staging system.

**Methods:**

Assessments were performed on 1125 patients with CVC between 2004 and 2015, and the results of those examinations were recorded in the Surveillance, Epidemiology, and End Results (SEER) database. Patients were randomly divided at a ratio of 7:3 into the training (n = 787) and validation (n = 338) cohorts. Predictors were identified using stepwise regression analysis in the COX regression model for create a nomogram to predict overall survival of CVC patients at 3-, 5-, and 8-years post-diagnosis. We compared the performance of our model with that of the AJCC prognosis model using several evaluation metrics, including C-index, NRI, IDI, AUC, calibration plots, and DCAs.

**Results:**

Multivariate risk factors including sex, age at diagnosis, marital status, AJCC stage, radiation status, and surgery status were employed to determine the overall survival (OS) rate (P<0.05). The C-index nomogram performed better than the AJCC staging system variable for both the training (0.737 versus 0.582) and validation cohorts (0.735 versus 0.573), which AUC (> 0.7) revealed that the nomogram exhibited significant discriminative ability. The statistically significant NRI and IDI values at 3-, 5-, and 8-year predictions for overall survival (OS) in the validation cohort (55.72%, 63.71%, and 78.23%, respectively and 13.65%, 20.52%, and 23.73%, respectively) demonstrate that the established nomogram outperforms the AJCC staging system (P < 0.01) in predicting OS for patients with cutaneous verrucous carcinoma (CVC). The calibration plots indicate good performance of the nomogram, while decision curve analyses (DCAs) show that the predictive model could have a favorable clinical impact.

**Conclusion:**

This study constructed and validated a nomogram for predicting the prognosis of patients with CVC in the SEER database and assessed it using several variables. This nomogram model can assist clinical staff in making more-accurate predictions than the AJCC staging method about the 3-, 5-, and 8-year OS probabilities of patients with CVC.

## Introduction

Ackerman discovered the uncommon and unique form of low-grade squamous cell carcinoma known as cutaneous verrucous carcinoma (CVC) in 1948 (1). This cancer develops slowly, is mostly exogenously, and keratoacanthoma-like tumors may appear anywhere on the surface of the skin. However, it appears most frequently on the plantar surface of the foot, anogenital area, and mouth. CVC is uncommon and has been found to occur on the face, maxillary antrum, and buttocks (2–4). Factors induced by chemical carcinogens, trauma, chronic irritability, and human papillomavirus are a few of the causes that have been linked to the development of CVC (5). Only a few instances of CVC have been documented to have metastasized to the local lymph nodes which cannot be attributed to skin metastases that have insufficient supporting documentation (6–9).

The incidence of CVC appears to be increasing rapidly, and it is now the second most common kind of skin cancer (10, 11). In the US, CVC constitutes 20% of skin cancers, corresponding to 1 million cases and contributing to up to 9000 predicted fatalities per year (12). Surgery is still the main treatment intervention. Radiotherapy and chemotherapy are adjuvant treatments, but for primary low-risk patients, the recurrence rate is 8–10% (13, 14).

The American Joint Commission on Cancer (AJCC) Staging Manual includes CVC, and is a significant tool for advising patients, selecting their best treatment, and categorizing them for clinical studies. However, there are a few important limitations in the AJCC staging system for CVC regarding factors that might not be assessed similarly across centers, such as differentiation grading. Another disadvantage is that no independent evaluation of histologic investigations has been performed, and hence risk factors are assumed to be missing if they are not reported. Some pathologic characteristics, such as the tumor depth in millimeters, are not recorded consistently and may impact some instances of AJCC staging. Furthermore, certain AJCC stages of CVC fall short of exact prognostic classification when outcome metrics differ (15–17).

Nomograms have emerged as a valuable predictive tool in the field of oncology in recent years (18). Compared to conventional evaluation methods, nomograms provide a more accurate and easily interpretable means of estimating the probability of a particular clinical outcome in an individual patient. This method has the potential to enhance the precision of prognostic assessments and facilitate more informed clinical decision-making (19). Nomograms have been widely utilized in the prognostication of various kind of malignancies (20–22), such as liver cancer, lung cancer, and breast cancer, their application in predicting clinical outcomes of patients with CVC remains inadequate. Presently, no predictive models have been established that can precisely prognosticate the overall survival (OS) of patients with CVC. Therefore, we have decided to investigate the survival rate of CVC utilizing Surveillance, Epidemiology, and End Results (SEER) data to aid clinicians and patients in determining appropriate treatment options.

The aim of this study was to establish a comprehensive nomogram for CVC patients using the Surveillance, Epidemiology, and End Results (SEER) database, which incorporates essential clinical and pathological features, demographic variables, treatment modalities, and other relevant characteristics. Consequently, the novel nomogram provides clinicians with more accurate and personalized patient survival predictions, superior to AJCC staging system in clinical efficacy. This is may have the potential to enhance population health by promoting improved quality of life and extending lifespan among patients.

## Materials and methods

### Patient source and extraction

We obtained patient data from the Surveillance, Epidemiology, and End Results (SEER) database, which includes 18 cancer registries and is publicly available at www.seer.cancer.gov. We used SEER*Stat version 8.3.6 software to retrieve and analyze the data. Additional access to the SEER Plus database was requested in compliance with ethical and legal standards. We reviewed information from the public SEER database, a cancer database that covers approximately 28% of Americans (23), and extracted data on CVC patients from this database (24). Subsequently, the proceeded as follows: the major CVC locations were chosen using the codes “C00.0 to C63.2.” All CVC-related ICD-O-3(third revision of the International Classification) histology and behavior codes (8051/3: Verrucous carcinoma, NOS) were included.

### Predictor selection

This study aimed to identify prognostic factors for cancer overall survival on CVC patients who were diagnosed between 2004-2015 and staged according to the sixth edition of the American Joint Committee on Cancer (AJCC) staging system which published in 2004. The various demographic and clinical variables screened as CVC prognostic factor that were age, sex, race, marital status, AJCC stage, surgery, radiation, cause-specific death, vital status, chemotherapy, tumor size, combined summary stage, and income. However, due to significant multicollinearity among these factors, we only used the AJCC staging system in the analysis. The outcome predicting variable was cancer overall survival. It is important to note that patient-informed permission was not necessary, as the SEER database used in this study did not include any personally identifying information.

### Data selection criteria

**Figure 1:**
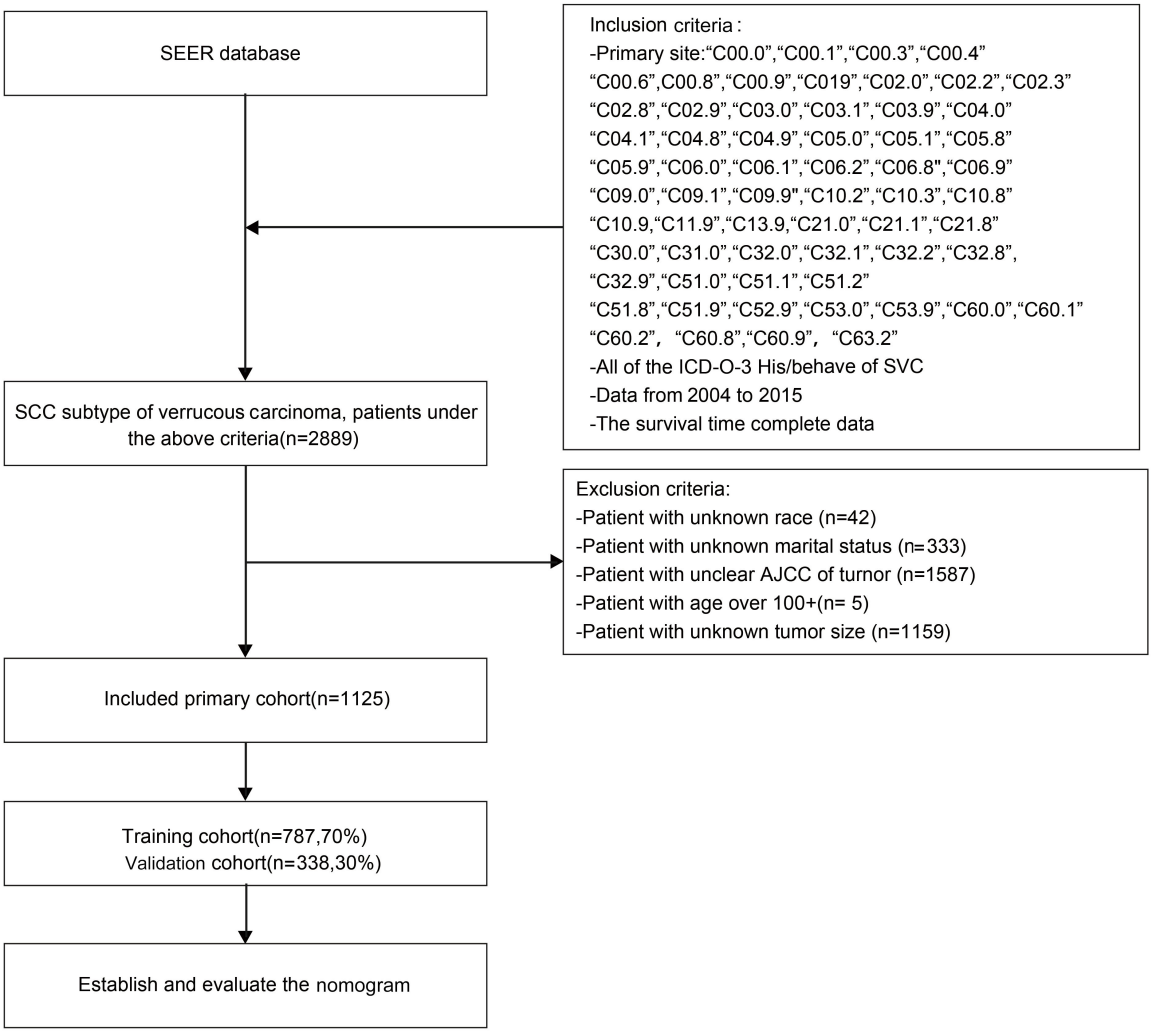
Flow chat of research selection. SEER, Surveillance, Epidemiology, and End Result Program; ICD-0-3, International Classification of Disease for Oncology, Third Edition.

A retrospective analysis was conducted using the SEER database, where 2889 patients with CVC between 2004-2015 were initially selected, based on the criteria mentioned previously. After a careful screening process, 1125 patients were finally selected, whereas 1764 patients were excluded due to unknown tumor size, race, or marital status, as well as unknown AJCC stage or an age exceeding 100 years old, which criteria were exclusion. The data selection procedure is depicted in **Figure 1**.

### Nomogram contruction and Cox regression analysis

In order to construct, 70% of patients were randomly allocated to the training cohort (n = 787) and 30% to the validation cohort (n = 338). We applied with univariate Cox regression to identify relevant prognostic factors, subsequent to multivariate Cox regression to determine independent risk factors in the training set. Hazard ratios (HR) and 95% confidence intervals (CI) were simultaneously recorded during this analysis. The nomogram was constructed based on a Cox regression model to identify significant variables for determining the 3-, 5-, and 8-year OS rates in patients with CVC.

Through the allocation of weights to each variable, multiple lines are created, with each variable corresponding to a specific point. Through the cumulative sum of points assigned to all variables, an overall score is obtained, which can be used to predict survival rates at different points in time.

### Nomogram verification and clinical applicability

A range of validation method were utilized to ensure the accuracy and reliability of the constructed nomogram in current study. The following describes the content and methods of evaluation applied in this study. These validation method was included the following texts mention:the calibration and discrimination of the nomogram were evaluated using bootstrapping with 500 resamples. Comparisons were conducted using net reclassification index (NRI) and integrated discrimination improvement (IDI), which are relatively new markers. This method made the comparisons more thorough and accurate. The concordance index (C-index) and the area under the time-dependent receiver operating characteristic curve (AUC) were employed as assessment tools to assess the potential for differentiation in the new model (25). The accuracy of the survival probability estimations made using the nomogram were evaluated using calibration plots. We further constructed judgment curves in order to assess the potential use of the nomogram in clinical contexts (26).

Decision curve analysis (DCA) is a novel method to assess the clinical utility of a model by determining the net benefit at different risk thresholds. DCA was employed for evaluating new nomogram of clinical potential application. The threshold probability and net benefit of the model were plotted on the abscissa and ordinate, respectively. A higher DCA curve for a model indicates greater clinical utility, as it reflects a higher net benefit at a given risk threshold (27).

### Statistical analysis

The software packages R (version 4.2.2; http://www.Rproject.org) and SPSS (version 25.0, SPSS, Chicago, Illinois, USA) were utilized for all statistical analyses of the data. In this analysis of 1125 patients, the log-rank test was used in R software to ensure that noticeable differences did not occur between the two cohorts. The continuous variable of age was quantified as median(25^th^-75^th^percentile) and did not follow a normal distribution. Percentages were used to express categorical variables.

The potential prognostic factors were identified using univariate Cox regression, and the relevant variables were included in the multivariate analysis. Then, a Cox regression model was selected using the stepwise regression method. A two-tailed test probability value of p < 0.05 was selected as the criterion for significance.

## Result

### Patient characteristics

This study comprised of 1125 patients with CVC, who were stratified into a training cohort (N=787) and a validation cohort (N=338). The clinicopathological and demographic characteristics of the two cohorts were summarized in **Table 1** using SPSS, and no statistically significant differences were found between the groups. The median ages at the CVC diagnosis were in 67 years (interquartile range (IQR), = 56–99 years) and 65 years (IQR = 54– 98 years) in the training and validation cohorts, respectively. Most of the patients in the training and validation cohorts were white (84.0% and 85.2%, respectively), married (52.2% and 51.2%), and male (59.2% and 59.8%). The AJCC cancer staging was in an early stage, predominately at stage I (49.3% and 50.9%), and local invasion predominated in both the training and validation cohorts (76.1% and 75.2%). Most patients accepted surgical resection treatment (89.8% and 88.8% in the training and validation cohorts, respectively) but refused radiotherapy (79.5% and 78.7%) and chemotherapy (91.5% and 90.5%). Upper-middle-income class families suffering from CVC in this study comprised about 42.2% and 41.7%, respectively.

**Table 1:** Patient characteristics and socio-demographic.

Variable	Training group	Validation Group
Number of patient n(%)	787(70)	338(30)
Diagnosis of age	67(56-99)	65(54-98)
Race n(%)		
White	661(84.0)	288(85.2)
Black	78(9.9)	27(8.0)
Others	48(6.1)	23(6.8)
Sex n(%)		
Male	466(59.2)	202(59.8)
Female	321(40.8)	136(40.2)
Married status n(%)		
Married	411(52.2)	173(51.2)
Single	153(19.4)	67(19.8)
Divorced/Separated/Widowed	223(28.4)	98(29.0)
AJCC staging n(%)		
I	389(49.3)	172(50.9)
II	240(30.5)	100(29.6)
III	99(12.6)	37(10.9)
IV	59(7.5)	29(8.6)
Combined Summary Stage n(%)		
Local	599(76.1)	254(75.2)
Regional metastasis	148(18.8)	66(19.5)
Distant metastasis	40(5.1)	18(5.3)
Surgery n(%)		
Yes	707(89.8)	300(88.8)
No/Unknown	80(10.2)	38(11.2)
Radiation n(%)		
Yes	161(20.5)	72(21.3)
No/Unknown	626(79.5)	266(78.7)
Chemotherapy n(%)		
Yes	67(8.5)	32(9.5)
No/Unknown	720(91.5)	306(90.5)
Income(US dollor) n(%)		
<$35,000, $35, 000-44,999	75(9.5)	38(11.2)
$45,000-$59,999	193(24.5)	78(23.1)
$60,000-74,999	332(42.2)	141(41.7)
$75,000+	187(23.8)	81(24.0)

### Variable screening

The significant variables in the univariate Cox regression analyses were age at diagnosis, AJCC stage, marital status, radiation status, sex, combined summary stage, tumor size, and surgery status, which were further assessed using multivariate Cox stepwise regression analysis(P<0.05). The following factors were significant after multivariate analysis, which results list in **Table 2**: age at diagnosis (HR = 1.059, 95%CI =1.050-1.067, p < 0.001), female (versus male: HR = 0.813, 95%CI =0.669-0.988,p = 0.037), divorced/widowed/separated (versus single: HR = 1.356, 95%CI =1.013-1.814,p = 0.040), AJCC stage II (combined summary stage versus AJCC stage I: HR = 1.224, 95%CI =1.001-1.496,p = 0.04), AJCC stage III (vs AJCC stage I: HR = 1.404, 95%CI =1.087-1.814,p = 0.010), AJCC stage IV (versus AJCC stage I: HR = 1.888, 95%CI =1.396-2.553,p < 0.001), without surgery (versus surgery: HR = 2.025, 95%CI =1.538-2.665, p < 0.01).

**Table 2:** Selected variables by multivariable Cox regression analysis.

	Multivariable analysis		
Variable	HR	95% CI	P-value
Diagnosis of age	1.059	1.050-1.067	<0.001
Sex			
Male	Reference		
Female	0.813	0.669-0.988	0.037
Marital status			
Single	Reference		
Married	0.814	0.621-1.067	0.137
Divorced/widowed/Separate	1.356	1,013-1.814	0.040
AJCC			
I	Reference		
II	1.224	1.001-1.496	0.04
III	1.404	1.087-1.814	0.010
IV	1.888	1.396-2.553	<0.001
Radiation			
Yes	Reference		
No/Unknown	0.843	0.668-1.063	0.149
Surgery			
Yes	Reference		
No/Unknown	2.025	1.538-2.665	<0.001

AJCC, American Joint Committee on Cancer; HR, hazard ratio, CI, confidence interval.

### Nomogram for OS prognosis

**Figure 2:**
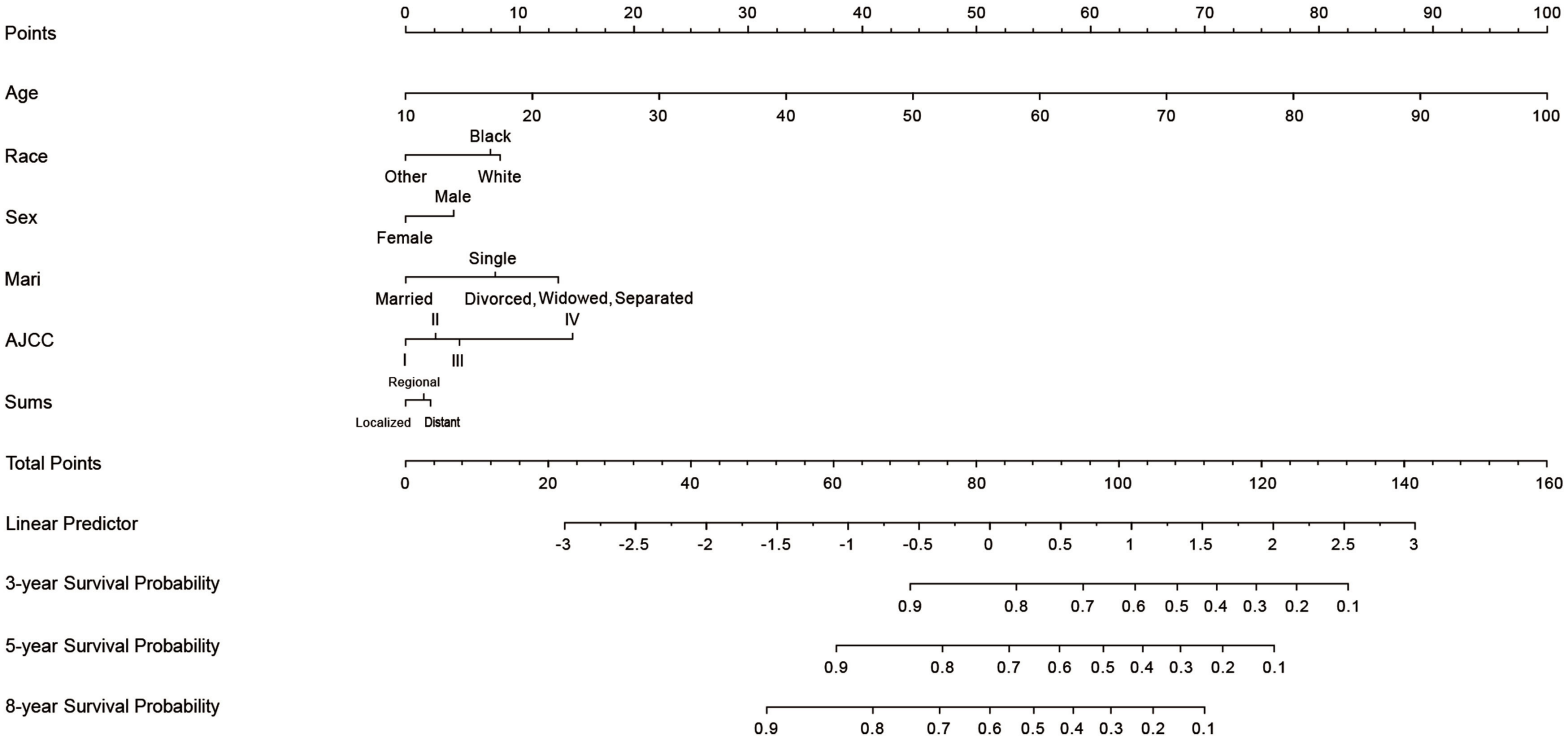
Nomogram for predicting 3-, 5-, and 8-years cutaneous verrucous carcinoma overall survival of probability. The value of each of variable was given a score on the point scale axis. A total score could be easily calculated by adding each single score and, by projecting the total score to the lower total point scale, we were able to estimate the probability of CVC. Sums, combined summary of stage; Mari, marital status; AJCC, American Joint Committee on Cancer.

A nomogram was constructed using chosen variables with their HRs, which comprised all the significant independent variables for forecasting the OS rates at 3, 5, and 8 years in the training cohort. **Figure 2** shows that age had the greatest effect on the prognosis according to the nomogram, followed by AJCC stage, marital status, race, sex, and combine summary stage(Sums). Each nomogram variable was given a score on a points system. After adding the scores for all variables, a line is drawn vertically downward to obtain the total score that indicates the OS probabilities at 3, 5, and 8 years.

### Evaluation of the OS nomogram

The C-indexes of the OS nomogram were 0.737 and 0.735, in the training and validation cohorts, respectively, compared with 0.582 and 0.573 for AJCC staging. Our model demonstrates superior discriminatory performance and prognostic ability compared to AJCC staging, as evidenced by its C-index values exceeding 0.7 and surpassing those of AJCC staging.

**Figure 3:**
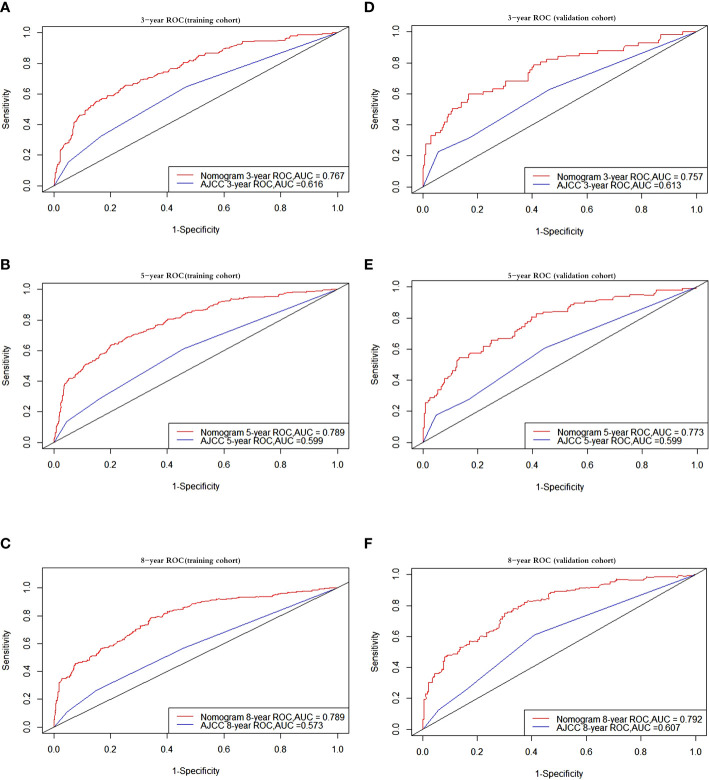
Receiver operating characteristic curves. ROC curve analyses to evaluate the performance of the new model compared to the traditional AJCC model. The area under the curve (AUC) metric was used to predict the overall survival probability with 3-, 5-, and 8-years OS probability in the training and validation cohorts. The results of the training cohort represent in **(A–C)** while **(D–F**) represent the results of validation cohort. OS, overall survival.

The AUC values for OS at 3, 5, and 8 years were 0.767, 0.789, and 0.789, respectively, in the training cohort, and 0.757, 0.773, and 0.792 in the validation cohort. The AUC was > 0.7 for the prediction of OS at 3, 5, and 8 years in both the training and validation cohorts (**Figure 3**), indicating favorable discrimination by the nomogram. The model demonstrated excellent discriminative capacity through its accurate predictions of the OS probabilities at 3, 5, and 8 years, which were made possible by highly precise predictive models of both set (**Figure 3**).

For the 3-, 5-, and 8-year OS probabilities, the NRI values were 68.08% (95% confidence interval [CI] = 0.559–0.867), 77.56% (95% CI = 0.677–0.945), and 79.34% (95% CI = 0.699–0.960), respectively, in the training cohort, and 55.72% (95% CI = 0.226–0.829), 63.71% (95% CI = 0.363–0.876), and 78.23% (95% CI = 0.561–0.964) in the validation cohort. The corresponding IDI values were 15.48%, 20.23%, 23.66%, 13.65%, 20.52%, and 23.73% (p = 0.001), respectively. When compared with the AJCC staging system, the new model performed much better in every circumstance in which the IDI and NRI values were higher than zero. These results indicating that the nomogram predicted prognosis with greater accuracy than the AJCC staging.

**Figure 4:**
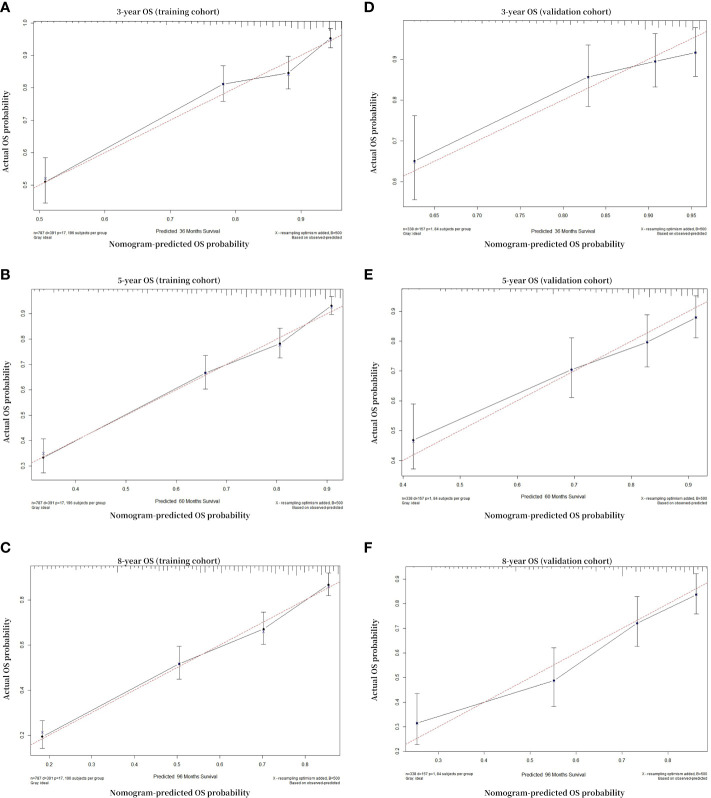
Calibration curves. Calibration curves for 3-, 5-, and 8-year OS depict the calibration of each model in terms of agreement between the predicted probabilities and observed outcomes of the training cohort **(A–C)** and validation cohort **(D–F)**. The solid black line indicates the ideal reference line where predicted probabilities would match the observed survival rates. The black dots are calculated by bootstrapping (resample: 500) and represent the performance of the nomogram. The closer the solid black line is to the dash red line, the more accurately the model predicts survival. OS, overall survival.

The calibration plot was used to test whether the model effectively differentiated between actual and expected values. The calibration curves of the nomogram showed high consistency between the predicted and observed survival probabilities in both the training and validation cohorts. In summary, the calibration plot of the OS nomogram demonstrated that the expected 3-, 5-, and 8-year survival probabilities for the training and validation cohorts closely matched the survival probabilities calculated using the actual data (**Figure 4**), indicating that the model had considerable discriminative and calibrating abilities. This proves that the model exhibited a high level of calibration.

**Figure 5:**
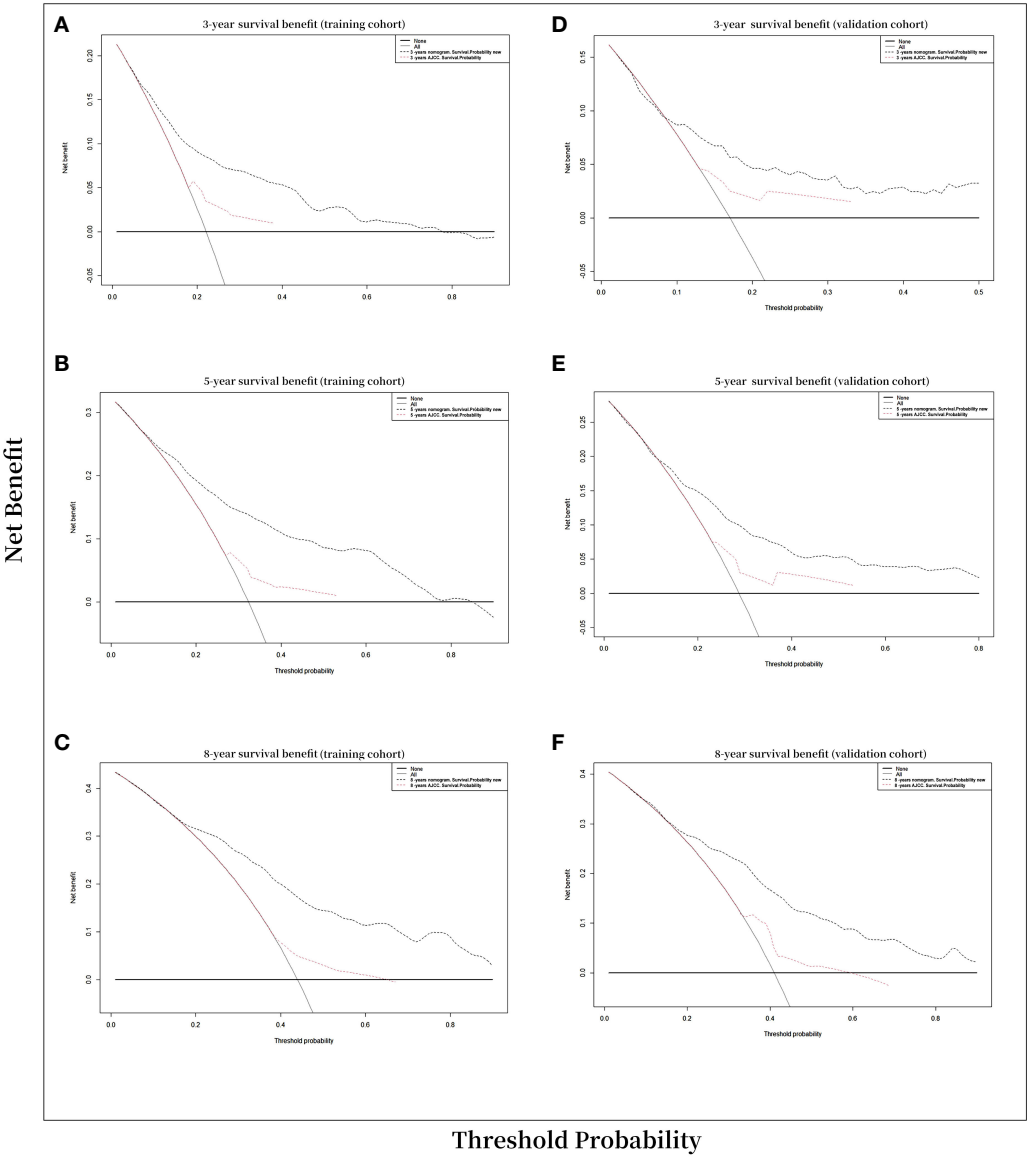
Decision curve analysis curves. Decision curve analysis of the nomogram for prediction of 3-, 5-, and 8-years overall survival probability with CVC patients. **(A)** 3-year survival benefit in the training cohort. **(B)** 5-year survival benefit in the training cohort. **(C)** 8-year survival benefit in the training cohort. **(D)** 3-year survival benefit in the validation cohort. **(E)** 5-year survival benefits in the validation cohort. **(F)** 8-year survival benefit in the validation cohort.

Ultimately, A decision-curve analysis (DCA) was conducted to assess the clinical validity of the new model, and satisfactory results were obtained for curves calculated at 3, 5, and 8 years in the training and validation cohorts (**Figure 5**). In the comparison between the clinical benefits of the nomogram and those of the AJCC staging, the DCA curves demonstrated that the nomogram outperformed the AJCC staging in predicting the 3-, 5-, and 8-year overall survival rates. This was evidenced by the fact that the nomogram provided more net benefits than the AJCC staging for nearly all threshold probabilities in both the training and validation cohorts.

## Discussion

With a lifetime prevalence of 7–11% in the USA, CVC is the second-most prevalent nonmelanoma skin cancer among white people (28). However, there are insufficient data for forecasting the OS in patients with CVC, and so our investigation addressed this aforementioned lack of research.

This study effectively used case data from the SEER database to construct a unique and simple prediction nomogram for patients with CVC. The 3-, 5-, and 8-year OS probabilities of patients with CVC can be estimated using this nomogram. In both internal and external evaluation, our nomogram showed satisfactory accuracy and discrimination. Nomograms can be used to identify and categorize participants in clinical trials to produce personalized prognostics. It is important for both the physician and the patient to properly interpret the probability of the 3-, 5-, or 8-year recurrence for the patient (29). We can examine two patients with CVC before AJCC stage IV as an illustration: a 60-year-old married white male known as patient 1 had a localized invasive tumor, while patient 2 was an 88-year-old black single female who had a distant metastasis tumor. The outcomes produced by the new nomogram were distinct: the 3-, 5-, and 8-year OS rate predictions for patient 1 were 80%, 68%, and 55%, respectively, and those for patient 2 were 28%, 12%, and 0%. We can identify patients with various prognoses using this nomogram, allowing for more-customized treatment and follow-up plans for this uncommon malignancy. The present results are consistent with several nomograms having been developed for other types of cancer that are more accurate than the current AJCC staging system (30).

Our study found that the average age at which CVC was diagnosed was 67 years, with males comprising the majority (>50%) of cases in both cohorts (**Table 1**). Multivariate analysis identified age as a significant risk factor for overall survival (OS), with older patients experiencing lower survival rates. Meanwhile, our model found that the nomogram score increased as the AJCC stage progressed, meaning that a higher AJCC stage was also linked to negative effects on patient survival (31). Females fared better than males in our study, and there were significant variations in OS related to sex, meaning that it is an important prognostic factor (**Table 2**). The healthy male and middle-aged population tendency may be related to alcohol use and health which has been documented.

It was particularly interestingly that this study found that having experienced divorce is a risk factor for the OS (HR = 1.356, p < 0.05) in CVC. A previous study found that divorced patients with cancer had worse outcomes than married patients (32), which may be related to a sudden interruption or loss of health insurance, reduction in social support, or financial instability, and raises the possibility that a patient may have worse outcomes after receiving cancer treatment (33). According to Hanske et al., the lower cancer screening rate among single people may be responsible for their higher risk of adverse outcomes (34). It can be speculated that unmarried or divorced patients undergo cancer screening less frequently, which could have an impact on their OS rate and cause more-advanced stages among this population. Previous research has found that supportive partnerships may have positive impacts on the behavior of a partner to obtain medical care (35).

Clinically, an CVC tumor lesion is defined by progression to a large, necrotic, and infected state with local aggressive metastasis, with results that are comparable to those of our study. Although uncommon, metastases to nearby lymph nodes and other distant regions are possible. Metastatic CVC has a fatal prognosis; our nomogram indicated that distant tumors in CVC increases negative outcomes for the survival rate, with a few large studies indicating mortality rates of 70% (36). This is an indication that the difficulty of treating metastatic CVC will depend on the affected areas and the degree of metastasis. Biologic aggression is well-documented and indicated by an increased frequency of numerous tumors, risk of local recurrence, regional and distant metastases, and higher mortality (37).

Our investigation found that patients who underwent surgery had an improved prognosis (**Table 2**). The outcome gives us a hint about CVC management: detecting tumors at an earlier stage is preferable since localized illness is frequently treatable with an appropriate surgical excision with sufficient margins (38), which may improve the survival probability and the prognosis of the patient. This might also facilitate a reduction in the frequency of local recurrences (39). Our study also found that receiving radiation as a monotherapy for CVC had no predominant effectiveness, while not receiving radiation was not a significant factor (**Table 2**). Although radiation therapy can shrink the tumor size, the patient who only receives radiation therapy may experience anaplastic transformation of their tumor that could eventually metastasize to the organ or the lymph node, which has been demonstrated in previous studies of skin cancer (6, 40). Radiation therapy can reduce the tumor size, but even with surgery, death might not be avoided.

A nomogram for OS has been constructed based on an assessment of the relevant prognostic indicators, and the nomogram was compared with the standard AJCC model by employing an internal validation cohort and a training cohort. The C-index and AUC were utilized to assess the discrimination abilities of the two approaches, and we discovered that both of these were superior for the monogram compared with the AJCC staging system in both the training and validation cohorts (**Figure 3**). An increase in AUC is not always immediately apparent when a brand-new metric is added to a model, and so a comparison needs to be performed to determine whether the predictive ability of the model has improved. Instead, the NRI is often used to compare the predictive capabilities of models, whereas the IDI may be used to indicate overall model progress (41, 42). According to the NRI of the nomogram model, the proportion of participants with correctly classified 3-, 5-, and 8-year survival probabilities increased after the new index was added by 68.08%, 77.56%, and 79.34% in the training cohort, respectively, and by 55.72%, 63.71%, and 78.23% in the validation cohort (p < 0.001). The IDI values indicated that the new model outperformed the AJCC staging system in terms of the probabilities of 3-, 5-, and 8-year survival by 15.48%, 20.23%, and 23.66% in the training cohort, respectively, and by 13.65%, 20.52%, and 23.73% in the validation cohort.

In order to establish the accuracy of our nomogram, the calibration curves and C-indexes were checked using both the training and validation cohorts. When subjected to internal and external validation, the C-indexes for the 3-, 5-, and 8-year OS models were 0.737 and 0.735, respectively. Both internal and external verification methodologies indicated that the C-index of the OS model exceeded 0.7. Excellent performance of the nomogram was also demonstrated by the calibration curves being highly consistent with the 45-degree ideal lines. These outcomes demonstrated that in both the training and verification cohorts, our nomogram had good calibration and discrimination performance (43) (**Figure 4**).

Decision-curve analysis (DCA) was employed to assess the clinical net benefit of the prediction models (44). The results of the study showed that the OS model had a beneficial impact on both the training and validation cohorts, as revealed by the 3-, 5-, and 8-year DCA curves, which demonstrated good performance (**Figure 5**). According to Vickers and Elkin (45), they have introduced DCAs to estimate the clinical utility of prediction models by determining the threshold probability, which is the probability at which the harm of a false-positive intervention exceeds the harm of a false-negative non-intervention, and subsequently derive the net benefit. In our current study, DCAs curve demonstrated significant net benefits for both the training and validation cohort. For example, in the validation cohort, assuming timely intervention for CVC patients with a 20% risk of mortality, every 6 and 15 of 100 individuals would benefit from the intervention at 3 and 5 years, respectively. The net benefits of clinical intervention were considered good when the threshold probability was greater than 0.4 at 3-,5-,8-year OS model (**Figure 5**).

While our study had important strengths, there were also a few limitations. First, because of the retrospective design of extracting data from the SEER database, selection and information biases were unavoidable. Second, therapy information in the SEER database is not all-inclusive; for example, no information was available on the type of surgical techniques utilized or some crucial clinical pathologic characteristics associated with prognoses, such as vascular invasion and the surgical margin. Third, the SEER database information lacks some laboratory tests results for important prognostic indicators, such as tumor and immunohistochemical analyses markers of p53, Rb gene, and HMB-45. Fourth, the projected values of the nomogram are only intended to serve as a general reference for doctors and will not always provide a correct prognosis. Future prospective studies will be conducted to test the nomogram to account for these limitations.

## Conclusion

This study is the first to utilize the SEER database to construct a comprehensive CVC nomogram based on an analysis of various variables. One intriguing finding was that divorce was a risk factor that harms the prognosis. Our nomogram may be useful as a tool to assist clinical staff in determining more-precise forecasts of the 3-, 5-, and 8-year OS rates of patients with CVC compared with using the AJCC staging system.

## Data availability statement

The original contributions presented in the study are included in the article/supplementary material. Further inquiries can be directed to the corresponding authors.

## Ethics statement

Ethical approval was not provided for this study on human participants because public database. Written informed consent to participate in this study was provided by the participants’ legal guardian/next of kin.

## Author contributions

SC and LH designed the study. JL and LD reviewed and edited the article. HY, HH, W-KM, CI, revised the article critically. H-HM, KL, XZ reviewed and edited the article. All authors approved the final manuscript.
